# Combating Emerging Respiratory Viruses: Lessons and Future Antiviral Strategies

**DOI:** 10.3390/vaccines12111220

**Published:** 2024-10-27

**Authors:** Palaniyandi Muthukutty, Jaime MacDonald, So Young Yoo

**Affiliations:** Institute of Nanobio Convergence, Pusan National University, Busan 46241, Republic of Korea; pmkpalani@gmail.com (P.M.); jaimemac.bgv@gmail.com (J.M.)

**Keywords:** respiratory viruses, pandemic, epidemic, coronavirus, influenza virus, antivirals

## Abstract

Emerging viral diseases, including seasonal illnesses and pandemics, pose significant global public health risks. Respiratory viruses, particularly coronaviruses and influenza viruses, are associated with high morbidity and mortality, imposing substantial socioeconomic burdens. This review focuses on the current landscape of respiratory viruses, particularly influenza and SARS-CoV-2, and their antiviral treatments. It also discusses the potential for pandemics and the development of new antiviral vaccines and therapies, drawing lessons from past outbreaks to inform future strategies for managing viral threats.

## 1. Respiratory Viruses: An Overview

Respiratory infections are a major global health concern due to their widespread prevalence, ease of transmission, and high morbidity and mortality, particularly in young children, the elderly, and immunocompromised individuals [1]. While often self-limiting and confined to the upper airways, these infections can cause severe lower respiratory tract diseases such as bronchiolitis and pneumonia in vulnerable populations: common cold (caused by rhinovirus, coronavirus, respiratory syncytial virus (RSV), and parainfluenza virus), bronchiolitis (caused by RSV), and pneumonia (caused by coronaviruses, RSV, and most influenza viruses) [2]. Viral transmission occurs through inhalation of airborne particles or contact with contaminated surfaces, leading to infection of respiratory mucosal cells. Seasonal and environmental factors, as well as zoonotic events, influence the incidence and severity of respiratory virus epidemics [3,4,5]. Understanding the transmission dynamics and host-pathogen interactions is crucial for developing effective prevention and treatment strategies.

Epidemiological studies indicate that seasonal and environmental factors, such as host susceptibility to temperature, humidity, host behavior, and immune tolerance, can influence respiratory virus epidemics [6,7]. Endemic strains of respiratory viruses (RVs), such as influenza and coronavirus, cause recurring seasonal infections; however, climatic changes and evolving animal trade and consumption can lead to zoonotic events, such as the highly pathogenic avian influenza A virus outbreaks and the recent SARS-CoV-2 pandemic (COVID-19) [8,9,10,11,12]. The interaction type and duration of contact with an infected animal determine zoonotic transmission efficiency, which can be spontaneous and sporadic. Human infection is determined by the availability of susceptible host receptors, which act as initial barriers against the highly evolved, multilayered human immune system [13,14]. When animal viruses that do not typically infect humans encounter the human immune system, they frequently trigger unbalanced activation of early innate immune response pathways [15,16]. Cell types expressing receptors that define the tissue tropism of the virus play a vital role in determining the severity and dynamics of viral infections [17]. Infections caused by highly pathogenic viruses can also result in systemic viral replication, secondary bacterial infections, and multi-organ damage [18].

Four past pandemics have been caused by RNA-based RVs, primarily influenza virus strains [19,20]. The 2009 H1N1 influenza virus pandemic and the 2019 coronavirus (COVID-19) pandemic are the most recent examples (Table 1). Given the antigenic mutations observed in both RVs, future pandemics seem inevitable [21,22]. To mitigate these threats, a thorough understanding of virology, genomic mutations, epidemiological characteristics, and immune evasion mechanisms is essential, and prevention strategies such as antiviral therapies must be developed to efficiently control RV outbreaks [23,24,25].

In this review, we comprehensively discuss current and potential future antiviral strategies for neutralizing emerging RVs, with particular focus on influenza and coronavirus.

## 2. Virology of Respiratory Viruses: Influenza and Coronavirus

Influenza viruses, part of the Orthomyxoviridae family, are classified into four types, namely, A, B, C, and D. Influenza A is responsible for the most severe illness and is known for causing seasonal epidemics and pandemics [30]. Since 1918, influenza A viruses have caused four pandemics, including the 2009 H1N1 outbreak. These viruses affect all age groups, with higher disease severity in infants, the elderly, and individuals with chronic health conditions [31,32,33]. Influenza viruses are enveloped, single-stranded, negative-sense RNA viruses with eight segments of negative-sense RNA that encode various proteins, including hemagglutinin (HA), neuraminidase (NA), nucleoprotein (NP), and matrix protein (M). The HA and NA proteins are crucial for determining host species tropism and are classified into subtypes based on the number of HA and NA variants. The HA (H1, H2, and H3) and NA (N1 and N2) subtypes are most frequently responsible for human infection and pandemics [34,35]. Influenza A virus is characterized by their high potential for antigenic shift and drift. Antigenic shift refers to the reassortment of viral RNA segments, while antigenic drift involves the gradual accumulation of point mutations due to the virus’s poor proofreading ability during replication [36,37]. Both processes contribute to the emergence of novel strains capable of escaping pre-existing immunity and causing future outbreaks.

Coronaviruses, belonging to the Coronaviridae family, are enveloped viruses with a positive-sense single-stranded RNA (ssRNA) genome, ranging in size from 26 to 32 kb, among the largest of all RNA viruses [38]. They derive their name from the crown-like spikes (glycoproteins) that protrude from their surface. These glycoproteins bind to specific receptors in humans and animals, facilitating cross-species transmission. Currently, seven coronaviruses are known to infect humans, causing symptoms ranging from the common cold to severe respiratory illnesses. Major recent epidemics caused by coronavirus include the 2003 Severe Acute Respiratory Syndrome (SARS-CoV), the 2013 Middle Eastern Respiratory Syndrome (MERS-CoV), and the 2019 SARS-CoV-2 (COVID-19) [39].

SARS-CoV-2 shares approximately 80% sequence similarity with SARS-CoV and about 50% with MERS-CoV [40,41]. It is a positive-sense RNA virus with 14 open reading frames encoding 27 proteins upon infection, including the spike (S), membrane (M), envelope (E), and nucleocapsid (N) proteins [42]. The spike protein, particularly its receptor-binding domain (RBD), interacts with angiotensin-converting enzyme 2 (ACE2) receptors on human cells, enabling the virus to enter host cells and initiate infection. SARS-CoV-2 also employs co-receptors such as transmembrane serine protease 2 (TMPRSS2) to facilitate viral entry into the host [43,44,45]. Viral ligand-cellular membrane receptor specificity determines the tropism of viruses to specific cells or tissues, which influences pathogenic symptoms. For cell infection to occur, a sufficient density of membrane receptors must bind to ligands on the virion surface [46,47,48].

Coronaviruses that cause severe respiratory diseases, such as SARS, contain spike proteins that interact with receptors like ACE2 on host cells. SARS-CoV-2 primarily targets lung and bronchial epithelial cells by binding to ACE2 receptors. The spike (S) glycoprotein consists of two subunits, S1 and S2, with the S1 subunit containing the receptor-binding domain (RBD). Furin, a proteinase, facilitates viral entry by cleaving the S1/S2 site of spike protein and mediates membrane fusion [49,50]. Other proteinases that facilitate virus entry, such as alanyl-aminopeptidase (ANPEP), glutamyl aminopeptidase (ENPEP), and transmembrane serine protease 2 (TMPRSS2), may also aid in the infection of lung cells [50,51,52,53,54].

As SARS-CoV-2 enters the respiratory system, it damages the tracheal mucosa. Symptoms include fatigue, fever, and coughing [55]. Severe infections lead to inflammation that damages the surrounding connective tissues, severely affecting the bronchioles and alveoli. The accumulation of inflammatory exudate in the alveoli hinders the exchange of oxygen and carbon dioxide, causing respiratory distress. This leads to further systemic damage, affecting the liver, kidney, neurological system, and other organs due to the lack of oxygen. Complications are exacerbated by a cytokine storm, which worsens the severity of infection [2,56,57].

## 3. Immune Response Against Respiratory Viruses: Influenza and Coronavirus

To develop an effective treatment regimen for viral respiratory infections, it is crucial to first understand the dynamics of systemic inflammatory responses. Individuals infected with RVs may be contagious one to two days before exhibiting symptoms and can remain infectious for several days after symptoms appear [58]. Immunocompromised individuals may remain contagious for longer than the typical infectious period [59,60].

Most RVs, including influenza and coronaviruses, trigger an immune response in the epithelial cells of the respiratory tract once infection occurs. In addition to epithelial cells, immune cells, such as alveolar macrophages and dendritic cells (DCs), play a vital role in detecting invading viruses through pattern recognition receptors (PRRs) [61]. These receptors recognize pathogen-associated molecular patterns (PAMPSs), initiating a cascade of signals that stimulate the production of various cytokines and chemokines. These inflammatory mediators alert innate immune cells, triggering a series of antiviral defense mechanisms. The innate immune recognition of viral infections activates gene expression that recruits inflammatory cells to the site of infection [62]. Early cytokines involved in the response are primarily members of the type I interferon family, including IFN-α and IFN-β [63]. Additionally, epithelial cells and alveolar macrophages produce chemokines, which recruit monocytes and T cells to the infection sites [64]. The coordinated expression of cytokines and chemokines leads to DC maturation, enabling antigen presentation to T cells in the draining lymph nodes. In summary, a localized inflammatory response, recruitment of innate immune cells, and cytokine-mediated signals influence DC maturation and migration to lymph nodes, where the adaptive immune response begins, playing a vital role in antiviral defense [65].

**Figure 1:**
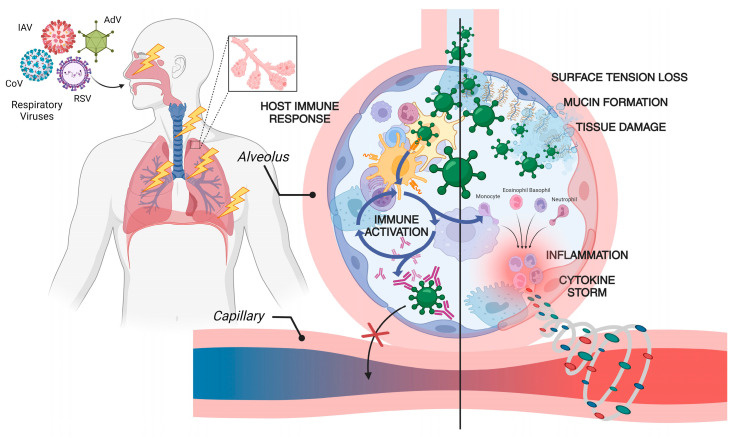
Respiratory virus infection, pathophysiology, and immune system activation. Once a respiratory virus enters the body via the nasopharyngeal route, viral particles penetrate the mucus and bronchial epithelial layer, damaging these cells and reaching the lungs, where replication occurs. Viral replication induces inflammation, leading to the activation of both the innate and acquired immune responses. An excessive immune response can result in the overproduction of large quantities of cytokines, including interferons (IFNs), interleukins (ILs), tumor necrosis factors (TNFs), transforming growth factors (TGFs), chemokines (CCLs), and C-X-C motif chemokines (CXCLs). This cytokine overproduction causes fluid leakage into lung cells, leading to hypoxia and severe lung damage, which can be fatal [71,72,73].

Following the activation of the innate immune system, DCs migrate to lymph nodes, where they stimulate CD4+ and CD8+ T cell activation via major histocompatibility complex (MHC) interactions [66]. The presence of naïve T cells specific to the antigen, along with co-stimulatory signals, results in the priming of T cells [67]. DC activation and maturation trigger the accumulation of virus-specific effector T-cells. T-cell receptor affinity, avidity, and co-stimulation from the local inflammatory environment influence the generation of differentiated effector T-cells, which then exit the lymph nodes and migrate to the lungs [68]. During the adaptive phase of the immune response, the expression of chemokines, chemokine receptors, and molecules associated with T cell trafficking increases [69]. Effector T cells in circulation recognize chemokines in infected areas, leading to changes in integrin affinity on blood vessel walls, facilitating extravasation into the virus-infected regions. As more effector T cells accumulate in the lungs, the viral load decreases, and infected cells are lysed [70]. A schematic illustration of RV infection through the respiratory tract and the subsequent inflammatory response that triggers a severe ‘cytokine storm’, elevating the disease’s severity, is shown in Figure 1.

## 4. Epidemiology and Transmission Dynamics: Influenza and Coronavirus

Respiratory droplets, aerosol transmission, and direct contact with contaminated surfaces are key human-to-human transmission pathways for both coronaviruses and influenza A viruses [74,75]. The risk of contracting these viruses is significantly higher when individuals are in close proximity to an infected person who is coughing, sneezing, or speaking [76,77,78]. Moreover, studies have detected viral particles in the feces of individuals infected with COVID-19 or influenza A, indicating potential for fecal–oral transmission [79,80,81]. Body secretions and fluids from confirmed cases are generally regarded as contagious [82,83]. Notably, influenza A has affected 9.3% of children aged 0 to 17, 8.8% of adults aged 18 to 64, and 3.9% of adults aged 65 and older [84]. In contrast, elderly individuals and those with underlying health conditions are the most vulnerable groups for COVID-19, with a higher probability of developing severe disease [85,86].

Some reports indicate that individuals infected with influenza A typically develop symptoms with one week of exposure and can be contagious one day prior to symptom appearance [87]. Notably, 80% of infected individuals may still be contagious up to five days after symptom resolution [88]. In the case of COVID-19, early studies revealed a median incubation period of approximately five days, with 97.5% of cases presenting symptoms within 11.5 days [89]. In rare cases, the incubation period has extended to as long as 16 days [90]. During the 1918 influenza A pandemic, an estimated one-third of the global population was infected, with approximately 50–100 million deaths attributed to the virus [91]. As of 2023, the World Health Organization reported that around 702 million people have been infected with COVID-19, resulting in 6.9 million deaths [92]. Currently, global vaccination efforts are considered the most effective strategy for controlling the spread of COVID-19 [93].

## 5. Antiviral Therapies for Respiratory Viruses: Influenza and Coronavirus

**Figure 2:**
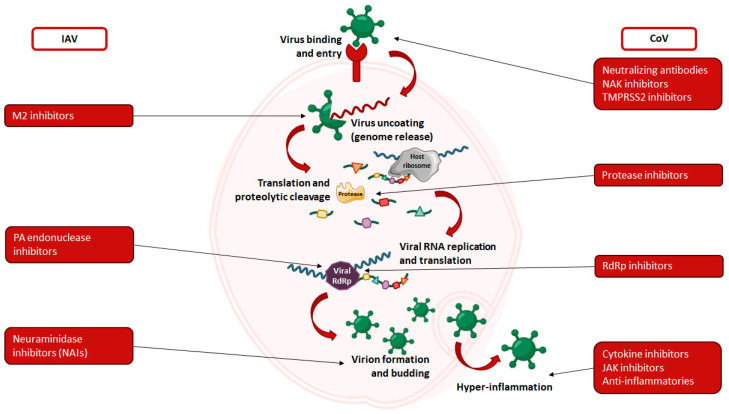
Schematic representation of the virus life cycle and inhibition strategies employed by antiviral drugs for influenza virus and coronavirus [95,96].

Antiviral agents are therapies designed to directly target a virus or its components to suppress its activity. This broad category includes a wide variety of therapies [94]. Antivirals typically interfere with viral entry or replication by disrupting the virus’s internal machinery (Figure 2). Common targets include viral RNA-dependent RNA polymerase (RdRp), proteases, and spike proteins. Each antiviral works through a specific mechanism to inhibit viral replication, either by disrupting the function of RdRp or reducing the efficiency of viral RNA replication. Targeting viral proteases, for example, interferes with the post-translational processing of viral progeny, which is essential for producing fully functional viruses. In addition, antiviral agents that target spike proteins usually bind to critical regions involved in viral attachment and entry into host cells. This process, known as viral neutralization, prevents the virus from entering and infecting cells, a topic that will be discussed in more detail in a separate section.

### 5.1. Antiviral Drug Options for Influenza Virus

Several antiviral drugs against the influenza virus have been developed and are approved by various regulatory authorities worldwide. These drugs target key stages of the viral infection and replication process, effectively inhibiting viral spread [97]. Some of the antivirals approved by the world drug authorities are listed in Table 2.

Baloxavir is an antiviral that prevents influenza infection by blocking the conversion of viral RNA to mRNA. This inhibition occurs when baloxavir captures the 5’ cap of pre-mRNA by binding and inactivating the polymerase enzyme, thereby halting viral replication.

Zanamivir (trade name Relenza) was the first neuraminidase inhibitor (NAI) approved by the FDA in 1999. Administered via intranasal or oral inhalation, zanamivir can reduce symptoms and lessen the need for antibiotics when taken at the onset of the infection [98]. Of note, zanamivir has poor oral bioavailability, making inhalation a more effective method for inhibiting viral replication [99,100].

Oseltamivir (trade name Tamiflu) is another NAI that prevents viral particles from binding to host cells, leading to functional inactivation of the virus and subsequent endocytosis by host cells [101].

Peramivir (trade name Rapivab) is a cyclopentane sialic acid analog that inhibits both influenza A and B viruses by targeting NA. This antiviral is notable for being the first intravenous NAI approved for use in critically ill patients. Peramivir binds more effectively to NA than other NAIs. It was approved for influenza treatment in Japan and Korea in 2010 and by the FDA in the United States in 2014. Several studies have demonstrated the safety and efficacy of peramivir [102,103].

**Table 2:** Summary of approved antiviral treatments for influenza virus.

Antiviral Agents	Virus Target	Mechanism of Action	Route of Administration	Category	Target	Indication	Approval Status	References
Favipiravir (Avigan^®^, Toyama Chemical, Tokyo, Japan)	Influenza A/B, SARS-CoV-2	RNA-dependent RNA polymerase inhibitor	Oral	Nucleoside analog	RdRp inhibitor	Investigational for COVID-19 and influenza	CT approved	[104]
Oseltamivir (Tamiflu^®^, Roche, Basel, Switzerland)	Influenza A/B	Neuraminidase inhibitor (prevents viral release)	Oral	Neuraminidase inhibitor	Neuraminidase	Treatment and prophylaxis of influenza A and B	FDA approved	[97]
Zanamivir (Relenza^®^, GSK, Middlesex, UK)	Influenza A/B	Neuraminidase inhibitor	Inhalation	Neuraminidase inhibitor	Virus budding inhibitor	Treatment of influenza A and B	CT approved	[105]
Peramivir (Rapivab^®^, BioCryst Pharmaceuticals, Durham, NC, USA)	Influenza A/B	Neuraminidase inhibitor	Intravenous (IV)	Neuraminidase inhibitor	Virus budding inhibitor	Acute, uncomplicated influenza	FDA approved	[106]
Baloxavir marboxil (Xofluza, Shionogi, Osaka, Japan)	Influenza A/B	Enzyme inhibitor, targeting the influenza virus’ cap-dependent endonuclease activity	Oral	Cap-dependent endonuclease inhibitor	RNA polymerase inhibitor	For individuals who are twelve years of age or older that have presented symptoms of this infection for no more than 48 h.	CT approved	[107]

CT: clinical trial; FDA: Food and Drug Administration.

### 5.2. Antiviral Drug Options for Coronavirus (SARS-CoV-2)

Based on previous experience with coronaviruses, such as SARS and MERS, antiviral medications have been employed since the early stages of the COVID-19 pandemic to disrupt the viral replicative cycle in patients. Current antiviral therapies target either viral proteins or host proteins that are essential for viral propagation.

Antivirals are most effective when administered during the early stages of the disease. For optimal outcomes, these treatments should be initiated within five days of symptom onset [108]. Delayed administration often results in reduced efficacy. Currently, several antiviral therapies have received emergency-use authorization (EUA) from the U.S. Food and Drug Administration (FDA) for COVID-19 treatment, including remdesivir, molnupiravir, and paxlovid (a combination of nirmatrelvir and ritonavir). Some of the approved antiviral drugs for SARS-CoV-2 are listed in Table 3.

#### 5.2.1. Viral RNA-Dependent RNA Polymerase (RdRp) Targeting Drugs

(a) Remdesivir

Remdesivir (Veklury^®^, Gilead Sciences, Foster, CA, USA) is an antiviral therapy initially developed to treat Ebolavirus and Marburg virus. It functions as a polymerase inhibitor, targeting the viral RdRp, an enzyme essential for viral RNA transcription. Since RdRp is highly conserved across coronaviruses, inhibiting this protein is a promising strategy for impeding viral replication. Remdesivir is a nucleoside analog of adenosine, which once metabolized into its active triphosphate form competes with ATP for binding with RdRp. This competition results in the incorporation of remdesivir into the nascent viral RNA strand. After these three nucleotides are added, RNA synthesis is prematurely terminated. The addition of these three nucleotides may also protect against viral exonucleases, further stalling the replication process [109]. As such, remdesivir acts as a chain-terminating nucleoside analog that halts RdRp activity [96].

While remdesivir is the most effective when administered early in the infection, studies have shown that it can also produce positive outcomes in later stages of severe COVID-19 infection. It is currently the only antiviral fully approved by the FDA for treating COVID-19. In contrast, most other antiviral treatments have only received EUA. The FDA has approved remdesivir for use in both hospitalized and non-hospitalized adults, as well as specific pediatric patients, particularly those at high risk of developing severe illness. Remdesivir has also demonstrated efficacy against emerging variants. Although meta-analyses have shown mixed results regarding its effectiveness, with some studies suggesting a protective benefit and others showing limited impact, remdesivir has generally improved clinical outcomes in hospitalized patients, though it has not significantly reduced mortality rates [110].

(b) Molnupiravir

Molnupiravir (trade name Lagevrio, Merck, Kenilworth, NJ, USA) is an RdRp inhibitor that limits viral replication and has shown activity against coronaviruses, including SARS-CoV-2. Unlike remdesivir, which causes stalling of RdRp, molnupiravir induces RNA mutagenesis, resulting in an increase in mutation frequency that prevents the formation of viable virions. As a nucleoside analog, molnupiravir is incorporated into the viral RNA during replication, leading to the accumulation of mutations that ultimately inhibit viral propagation [111]. A meta-analysis of clinical trials demonstrated that approximately 95% of patients showed clinical improvement by day 5 of treatment with molnupiravir [112]. In December 2021, the FDA granted EUA of molnupiravir to adults with mild-to-moderate COVID-19 who are at high risk of progressing to severe disease resulting in hospitalization or death and for whom no other treatment options are available or appropriate. For maximum efficacy, treatment with molnupiravir should be started immediately after COVID-19 diagnosis and within five days of symptom onset. Currently, molnupiravir is recommended for patients with mild COVID-19 who are at a high risk of hospitalization [113,114,115].

#### 5.2.2. Other Antiviral Drugs

(a) Favipiravir

Favipiravir is a modified pyrazine analog originally approved for the treatment of Ebola and influenza viruses. Like remdesivir, favipiravir acts as a nucleoside analog that inhibits RdRp by being incorporated into nascent viral RNA strands, leading to chain termination. The incorporation also results in a high mutation rate that reduces the production of viable virions [116]. While favipiravir can shorten the duration of viral clearance, it has limitations due to the exonuclease activity of viral RdRp, which allows for proofreading and mutation repair. Favipiravir is not recommended in the latest international treatment guidelines for COVID-19 [104,117,118].

(b) Ribavirin

Ribavirin is a synthetic nucleoside analog of ribofuranose, primarily used for RNA viruses, including the hepatitis C virus. It alters the viral genome by integrating into the viral RNA, preventing proper replicating. Ribavirin binds to viral RdRp with an affinity comparable to native nucleotides. However, it is not used to treat COVID-19 due to its unfavorable side effects and low efficacy [119,120].

Protease enzymes are essential for viral protein maturation as they cleave translated viral polypeptides into functional proteins, making them potential targets for viral therapy. Studies have explored the potential use of protease inhibitors for COVID-19, focusing on the SARS-CoV-2 major protease (M-pro or 3CL-pro), which is essential for viral replication. Molecules that bind M-pro can disrupt its catalytic activity, reducing viral replication [121].

(c) Lopinavir/Ritonavir

Lopinavir is a protease inhibitor initially developed for HIV-1 treatment and is currently used in combination with ritonavir for HIV-AIDS. The combination of lopinavir and ritonavir was also tested for COVID-19. Although this combination alone has proven ineffective in treating COVID-19 patients, it may provide benefits when combined with other therapies [122]. Lopinavir specifically inhibits SARS-CoV-2’s M-pro, avoiding off-target effects. Ritonavir boosts the effectiveness of lopinavir by inhibiting cytochrome P450 (CYP3A4), which extends the half-life of lopinavir and enhances its suppression of SARS-CoV-2 replication. However, evidence supporting the efficacy of lopinavir/ritonavir in COVID-19 treatment is insufficient, and current WHO guidelines do not recommend its use [121,123].

(d) Nirmatrelvir/Ritonavir (Paxlovid)

Nirmatrelvir is a potent M-pro inhibitor, originally developed to combat SARS-CoV-1. The FDA approved the use of this combination (under the trade name Paxlovid, Pfizer, New York, NY) for treating COVID-19 [124]. Studies have shown that nirmatelvir/ritonavir reduces hospitalization and mortality rates in COVID-19 patients, although further research is needed to confirm its efficacy. Currently, the WHO guidelines recommend nirmatrelvir/ritonavir for COVID-19 treatment, as it may be more effective than other options in preventing severe disease and hospitalization [125,126].

#### 5.2.3. Convalescent Plasma

Convalescent plasma therapy was introduced in the 1890s by Emil Von Behring and Shibasaburo Kitasato as a treatment for infectious disease [127]. This approach leverages the role of antibodies in neutralizing antigens, with the passive administration of pathogen-specific polyclonal or monoclonal antibodies (mAbs) preventing viral entry into host cells, thus inhibiting infection. Due to the initial lack of approved and effective therapies for COVID-19, convalescent plasma was one of the first treatments explored during the early stages of the pandemic. Historical use of convalescent plasma (obtained from individuals who recovered from viral infections) dates back to the 1918 flu pandemic [127].

Shen et al. reported the use of convalescent plasma transfusion in five critically ill patients with COVID-19, resulting in clinical improvements and viral neutralization [128]. In August 2020, the U.S. FDA approved and granted EUA for the use of convalescent plasma to treat hospitalized patients with COVID-19 [129]. Most clinical studies investigating this therapy have found that convalescent plasma is primarily associated with only minor transfusion-related adverse events; this result is despite conflicting data on its effectiveness in patients with COVID-19 in terms of the time of administration during the course of the disease, number of infusions required, and optimal titers. Several clinical studies have reported no significant improvement in hospitalized patients with COVID-19. However, patients can benefit from early infusion of high-titer convalescent plasma [130,131]. Ongoing clinical trials continue to explore the potential effectiveness of convalescent plasma transfusion as a COVID-19 therapy [132,133].

#### 5.2.4. Monoclonal Antibodies

mAbs are merged as effective therapeutic agents for specifically targeting SARS-CoV-2, as they help restore immunological balance by inducing a rapid, passive immune response. This response aids in the destruction of infected cells and reduces the viral load [134]. Many mAbs have received emergency authorization for the treatment of individuals with mild-to-moderate COVID-19, showing significant efficacy in lowering hospitalization and death rates [135].

SARS-CoV-2 enters the host cells via the ACE2 receptor, which is highly expressed in the respiratory tract. To block viral entry, most mAbs are designed to target epitopes on the viral spike (S) glycoprotein, specifically the S1 and S2 subunits. Since spike protein exhibits high mutation rates, the efficacy of mAbs depends on the circulating variant. Bamlanivimab, casirivimab, and imdevimab were the first mAbs approved by the FDA for emergency use, and they have been shown to reduce hospitalization and mortality compared to placebo [136,137,138,139]. Administering these mAbs in combination rather than individually has resulted in more positive clinical outcomes and offers a strategy for overcoming resistance [140].

Clinical trials have demonstrated that the combination of casirivimab and imdevimab (REGEN-COV), when administered within five days of infection, can reduce hospitalization by approximately 56.4%, decrease disease severity by 59.2%, and prevent death by 93.5% over a 28-day treatment period [138,141]. However, the emergence of new variants, such as Omicron and its sublineages, has led to resistance against many of these early mAbs, resulting in the withdrawal of their EUA.

Recently, bebatelovimab has become important owing to its efficacy against all known COVID-19 variants, including Omicron, prompting EUA by the FDA and pending approval by the European Medicines Agency (EMA) [142,143]. While mAbs provide considerable protection and positive recovery outcomes, the ongoing emergence of COVID-19 variants requires careful consideration of the potential for resistance in the context of a rapidly evolving pandemic [144,145].

Although influenza and coronaviruses are RVs that primarily infect the respiratory tract, their extrapulmonary effects can impact the gastrointestinal, cardiac, neurological, and endocrine systems. It is essential to determine whether these effects are caused directly by viral infection, inflammation, or an immune system-based cytokine storm before optimizing antiviral therapies.

Ongoing surveillance of RVs, along with comprehensive studies, can help mitigate future epidemics or pandemics. By curbing the transmission and implementing strategic medical countermeasures, such as a combination of antivirals and vaccines, the human immune system can be better equipped to handle emerging treats.

**Table 3:** Summary of approved antiviral treatments for Coronavirus.

Antiviral Agents	Virus Target	Mechanism of Action	Route of Administration	Category	Target	Indication	Approval Status	References
Remdesivir (Veklury^®^)	SARS-CoV-2	Inhibits RNA-dependent RNA polymerase (RdRp)	Intravenous (IV)	Adenosine analog	RdRp inhibitor	Hospitalized and non-hospitalized adults and pediatrics at high risk of progression to severe disease	FDA approved	[146]
Molnupiravir (Lagevrio^®^)	SARS-CoV-2	Induces viral RNA mutagenesis	Oral	Nucleoside analog	RdRp inhibitor	Adults with mild-to-moderate COVID-19 are at high risk of progression to severe disease	EUA and approved in many countries	[147]
Nirmatrelvir + Ritonavir (Paxlovid^®^)	SARS-CoV-2	Protease inhibitor (inhibits viral replication)	Oral	Protease inhibitor	Mpro	Mild-to-moderate COVID-19 patients at risk of progression to severe disease	Approved in the United States, the United Kingdom, and EU; EUA in many countries	[148]
Bebtelovimab	SARS-CoV-2 (all variants)	Monoclonal antibody (targets spike protein)	Intravenous (IV)	mAb	S-protein	Treatment of COVID-19 in non-hospitalized patients	EUA by US FDA	[143]
Sotrovimab (Xevudy)	SARS-CoV-2	Monoclonal antibody (targets highly conserved sequences)	Intravenous (IV)	mAb	S-protein	Mild-to-moderate COVID-19 patients at risk of progression to severe disease	EUA or approved in many countries	[149]
Casirivimab and imdevimab (REGEN-COV)	SARS-CoV-2	Monoclonal antibody (targets spike protein)	Intravenous (IV) and subcutaneous (SC)	mAb	S-protein	Mild or moderate COVID-19, conditional approval for the prophylaxis and treatment of acute COVID-19 in the United Kingdom	EUA in many countries	[141]
Ensitrelvir (Xocova)	SARS-CoV-2	3C-like protease inhibitor	Oral	Small molecule	Mpro	May be effective in treating smell and taste loss from the COVID-19 infection	Approved in Japan	[150]
Simnotrelvir + ritonavir (Xiannuoxin)	SARS-CoV-2	Protease inhibitor	Oral	Small molecule	Mpro	Mild-to-moderate COVID-19	Approved in China	[151]
VV116	SARS-CoV-2	Nucleoside analogue antiviral drug	Oral	Small molecule	RdRp inhibitor	Non-hospitalized adults with mild-to-moderate disease	Approved in China	[152]

EUA: Emergency Use Authorization.

## 6. Clinical Studies and Approval of Antiviral Treatments for RVs

To reduce mortality rates and combat RV infections, it is essential for medical professionals and scientists to develop effective antivirals and vaccines. A major challenge in this endeavor is the need for continuous monitoring and distribution of drug supplies through an expansive global network of institutions, especially as disease rates fluctuate globally. This necessitates collaboration among healthcare providers, pharmaceutical companies, and government bodies to ensure that treatments are both effective and accessible. Table 4 provides a summary of the approved antiviral treatments as well as ongoing clinical trials targeting RV infections, highlighting the latest progress in combating these diseases.

## 7. Antiviral Vaccines for Respiratory Virus: Influenza and Coronavirus

Vaccination’s two primary purposes are to protect against infectious diseases and to stimulate the immune system. However, the efficacy and effectiveness of vaccines vary depending on the type of infection and the specific product. Historically, traditional vaccines were developed by inactivating or attenuating the corresponding pathogen, which effectively curtailed the spread of infections and contributed to the eradication of diseases. However, there are still concerns with conventional vaccines, such as the inclusion of unwanted contaminants, structural alterations in protein-laden antigens, and the possibility that inactivated antigens may not always elicit a strong immune response [153,154,155].

In a pandemic crisis, producing billions of safe and effective vaccine doses within a shorter timeframe poses significant scientific challenges, which hinge on three key factors. First, selecting the appropriate scientific approach to produce absolute immunogenicity is essential for the complete eradication of viral reservoirs. Second, an effective platform must be available for mass production of vaccines. Third, the timely distribution of vaccines to all regions of the world is necessary. The primary goal of vaccine development is to create the safest vaccines that elicit a robust immune response. Achieving this goal depends on unprecedented cooperation between industries, researchers, and regulators [156,157,158].

Vaccination is the sole course of action for achieving herd immunity, which is crucial in halting the spread of viruses during epidemics or pandemics [155]. Under normal circumstances, vaccines take several years to develop, with only about 10% of vaccines receiving authorization for commercialization. Typically, clinical development and commercialization of vaccines take five to ten years [159]. However, recent pandemics have placed immense socioeconomic pressure on accelerating vaccine development from classical approaches to more novel nucleic acid-based vaccines [155].

Before developing a vaccine, certain criteria must be carefully evaluated, starting from target antigen detection, mode of administration, animal models, scale-up, production, manufacturing, storage, and transportation [160]. Selecting the right vaccine type—along with the appropriate adjuvants, excipients, dosage, optimal route of administration, and booster doses—can significantly improve vaccine effectiveness against RV infections [161]. Traditional vaccines include inactivated viruses, live attenuated vaccines, protein subunit vaccines, and virus-like particles, whereas new-generation vaccines include recombinant viral vector vaccines, nucleic acid-based vaccines, and antigen-presenting cell-based vaccines [162]. Each class of vaccines has its own advantages, including genetic manipulation, strong immune response, prolonged stability, and convenience for large-scale manufacturing.

### 7.1. Vaccine Types

#### 7.1.1. Whole Pathogen Vaccines

Conventional vaccines against whole pathogens comprise either inactivated/killed or attenuated/live pathogens [163]. Attenuation significantly reduces the viral virulence and replicability of pathogens. However, in rare cases—particularly in immunocompromised patients—attenuated whole pathogen vaccines can cause infection, exhibit toxicity, or trigger unwanted immune responses [164]. Inactivated vaccines contain whole viruses that have been destroyed and are unable to replicate. As a result, they do not constantly stimulate the immune system, which may necessitate multiple doses and/or the use of an external adjuvant to elicit the desired immune response [165]. One drawback of using whole viruses is that they may produce infectious particles or degrade viral antigens and epitopes during the inactivation process, potentially reducing their effectiveness [166].

#### 7.1.2. Subunit Vaccines

Subunit vaccines are composed of specific virus components, such as proteins, peptides, or polysaccharides, which can trigger protective immune responses [167]. It has been shown that the particle size in subunit vaccine formulations plays a critical role in determining vaccine efficiency [168]. Proteins, nucleic acids, and polysaccharides can all be employed as antigens. Subunit vaccines are safer for use in immunocompromised individuals and carry a lower risk of adverse effects compared to whole pathogen vaccines. However, due to the smaller size of the antigenic components, they may generate lower immunogenicity, necessitating the use of adjuvants and specialized delivery systems to enhance immune responses [169].

#### 7.1.3. Nucleic Acid Vaccines

DNA vaccines contain target protein-encoding DNA and delivery vectors that allow vaccine components to reach the cell nucleus, where DNA is translated into mRNA for protein biosynthesis. DNA vaccines have the potential to stimulate both B cells and T cells in the immune system, but delivering DNA into the nucleus remains a significant challenge in the development of these vaccines. Recent advancements have focused on optimizing DNA constructs and delivery vectors to improve the efficacy and safety of DNA vaccines [170,171,172].

Another novel approach to vaccination is the use of genetic material encoding the antigen(s) against which an immune response is desired [173]. The genetic material is introduced into human cells, which then produce target proteins that serve as antigens to elicit an immune response. A notable recent development in the field is the mRNA vaccines [174,175,176]. This strategy has demonstrated significant promise, particularly in the contest of the COVID-19 pandemic [177,178].

### 7.2. Viral Vaccines Combating Influenza Virus

Of all RVs, the influenza virus not only causes seasonal episodes but also triggers epidemic and pandemic outbreaks, leading to significant illness and fatalities worldwide. Over the last century, there have been four major influenza-related pandemics: the 1918 A/H1N1 pandemic, often referred to as the “Spanish Flu”, the 1957 A/H2N2 reassortment of human and avian viruses originating in China, the 1968 A/H3N2 influenza that struck Hong Kong, and the most recent A/(H1N1) pdm09 variant that emerged in North America in April 2009 [179]. Each of these four pandemics resulted in at least 10,000 deaths, underscoring the urgent need for the development of universal influenza vaccines [180].

Seasonal influenza epidemics cause an estimated 290,000 and 650,000 respiratory-related deaths and between 3 and 5 million cases of severe illnesses globally each year [181]. Influenza epidemics, commonly referred to as the seasonal flu, are caused by novel influenza strains that emerge intermittently every two to five years. These outbreaks, which may be influenced by weather and climate, can last from three to six months depending on the region [182,183,184]. The influenza virus is able to evade the human immune system through unique mutations in its surface glycoproteins, HA and NA [185]. This is why neither spontaneous infection nor vaccination can provide lifelong immunity against the virus [186].

Currently, three types of influenza vaccines are used globally: recombinant HA vaccines, live attenuated influenza vaccines (LAIVs), and inactivated influenza vaccines (IIVs) [187]. Due to the limited duration of immunity and the need to account for the antigenic variations of circulating viruses, all influenza vaccinations need to be updated annually [188].

#### 7.2.1. Inactivated Influenza Vaccine

The inactivated viral vaccine, which holds the largest proportion in the global flu vaccine market, is widely used due to its high safety and comparatively low production costs [189]. This type of vaccine is typically produced by cultivating the virus in cultured mammalian cells or embryonated chicken eggs [190,191]. Inactivated influenza vaccines can induce both systemic and local immunity, yet booster shots may be required to maintain adequate antibody titers [192]. These vaccines can be further categorized into three types based on production techniques: whole-virus inactivated vaccine, split-virus inactivated vaccine, and subunit inactivated vaccine [187]. Split-virus and subunit vaccines are more frequently used than whole-virus vaccines due to their similar immunogenicity and reduced risk of adverse reactions [193].

#### 7.2.2. Live Attenuated Influenza Vaccines

LAIVs stimulates both humoral and cellular immunity by mimicking natural infection and immunization without causing significant harm to the recipient [194,195]. These vaccines use a temperature-sensitive, cold-adapted virus strain that replicates preferentially in the nasopharynx but is unable to reproduce efficiently in the warmer environment of the lower respiratory tract [196,197]. However, due to the risk associated with live viruses, LAIVs are not recommended for immunocompromised individuals or those in close contact with them [187].

#### 7.2.3. Recombinant HA Vaccine

Recombinant HA vaccines are produced by cloning the DNA encoding HA into a vector, such as a baculovirus, and generating recombinant viruses in insect cells. This method offers high production capacity at low cost [198,199,200]. A key advantage of recombinant HA vaccines is that they avoid unwanted mutations from egg adaptation, making them a preferable option for individuals allergic to eggs [200]. While the mechanism of action for the recombinant HA vaccine is similar to that of the inactivated influenza vaccine, the recombinant HA vaccine requires only one-third of the HA to elicit the same antibody response due to its higher immunogenicity [201]. Additionally, recombinant HA vaccines are most suitable for pandemic preparedness because they can be produced faster and are safer against highly pathogenic viruses. However, they are currently only approved for use in adults, as they have been found to be ineffective in children [202].

Regular vaccination against influenza and its variants is essential, as immunity wanes over time. Annual influenza vaccination helps maintain protection. The effectiveness of the seasonal vaccine depends on how well the vaccine strain matches the circulating virus strain [203,204]. Although limitations exist regarding vaccine effectiveness, advancements in vaccine technologies and increased production of more efficacious vaccines have helped reduce the burden of disease and prevent outbreaks [153]. A list of various influenza virus vaccines approved for commercialization is presented in Table 5.

### 7.3. Viral Vaccines Combating Coronavirus

The recent SARS-CoV-2 pandemic created a global health crisis, overwhelming healthcare systems in many countries for several months. In response, vaccines became urgently necessary to reduce fatalities and achieve herd immunity [205]. The collaboration between academic institutions, biotechnology companies, pharmaceutical firms, and government organizations led to the EUA of several vaccines, which demonstrated credible safety and efficacy in preventing the spread of the disease. These EUA-approved vaccines played a crucial role in mitigating the pandemic and reducing loss of life [155].

According to the latest WHO reports, more than 50 COVID-19 vaccines of various genres have been approved, 183 vaccine candidates are being tested in clinical trials, and 199 are in the preclinical stage [206].

#### 7.3.1. Inactivated Whole Virus Vaccines

The traditional approach to developing a whole virus vaccine involves administering an inactivated or live attenuated form of the virus [207]. For inactivated whole-virus vaccines, the SARS-CoV-2 cultivated in cells is inactivated using agents such as formaldehyde or glutaraldehyde or through exposure to UV or gamma radiation [208]. As of right now, 11 inactivated COVID-19 vaccines have received approval across the globe [209].

The Covilo vaccine, developed by Sinopharm (Beijing, Chia), is the most widely authorized inactivated COVID-19 vaccine, receiving approval in 93 countries. The vaccine contains two SARS-CoV-2 strains, WIV04 and HB02, grown in the Vero cell line and inactivated using β-propiolactone. Aluminum hydroxide is used as an adjuvant. The estimated vaccine efficacy against the symptomatic COVID 2019 was 72.8% for WIV04, 78.1% for HB02, and 79% against severe illness or hospitalization [210,211,212].

Another inactivated vaccine, CoronaVac, was developed by Sinovac Life Sciences Co. (Beijing, China); this vaccine uses the SARS-CoV-2 strain CN02, also cultured in Vero cell line and inactivated with β-propiolactone. Like Covilo, it contains aluminum hydroxide as an adjuvant to boost the immune response. CoronaVac has been approved in 56 countries, and its efficacy varied from 50% in symptomatic patients to 83.5% in asymptomatic patients [213].

In India, Covaxin^®^ (BBV152), developed by Bharat Biotech (Hyderabad, India), was the second-most frequently administered vaccine. It is a β-propiolactone inactivated vaccine, incorporating the whole virion along with an adjuvant made of imidazo-quinoline gallamide adsorbed on alum, which enhances vaccine antigen delivery to lymph nodes without causing systemic influx. The vaccine is based on the genetically stable strain NIV-2020-770, which contains the Asp614Gly mutation [214]. Covaxin demonstrated inconsistent seroconversion rates, reaching 90% in cohorts tested closer to the Omicron wave [215]. The vaccine stimulates follicular T helper cells, which aid in boosting B cell responses, generating strong immunological memory against SARS-CoV-2 and other variants of concerns [215].

The Chumakov Centre (Moscow, Russia), part of the Russian Academy of Sciences, developed the KoviVac vaccine, which has been approved in Russia, Belarus, and Cambodia. KoviVac uses the SARS-CoV-2 strain AYDAR-1, inactivated with β-propiolactone, with aluminum hydroxide as an adjuvant. In clinical trials, seronegative participants showed an 86.9% seroconversion rate. The vaccine was found to have good safety and tolerability [216,217].

#### 7.3.2. Protein Subunit Vaccine

Immunizations against a wide range of infections are produced using protein subunits made up of one or more distinct viral antigens [218]. These vaccines usually require an appropriate adjuvant when administered to humans to trigger a robust immune response. Subunit vaccine formulations combine purified antigens with powerful adjuvants to enhance immunogenicity [219]. As of now, 19 protein-based subunit COVID-19 vaccines have received global approval [209].

Novavax (Gaithersburg, MD, USA) developed the NVX-CoV2373, also known as Nuvaxovid, which is the most widely authorized protein subunit COVID-19 vaccine, approved in 40 countries. Nuvaxovid is a recombinant DNA product made in an insect cell line derived from Sf9 cells of the *Spodoptera frugiperda* species. It contains the S protein from the original SARS-CoV-2 virus strain. The vaccine also includes Matrix-M^TM^, a proprietary saponin-based adjuvant, and polysorbate 80 (PS80) to stabilize nanoparticles. In clinical trials conducted in the United States and Mexico, the vaccine showed efficacy of 90.4%, while trials in the United Kingdom demonstrated an efficacy of 89.7%. Adverse reactions, mostly mild to moderate, were frequent after the second dose and typically resolved within days [209,220,221].

Another subunit COVID-19 vaccine, VidPrevtyn Beta, was developed by Sanofi (Lyon, France)/GSK (Rixensart, Belgium) and has been authorized in 30 countries. This vaccine uses the spike protein derived from the Beta variant (B.1.351 strain) of SARS-CoV-2, produced using recombinant DNA technology through a baculovirus expression system in insect cells from *Spodoptera frugiperda*. To boost the immune response, AS03, a combination of squalene, DL-α-tocopherol, and polysorbate 80, is used as an adjuvant. Comparative research indicated that the VidPrevtyn Beta booster showed efficacy comparable to the mRNA vaccine, including against the Omicron BA.1 subvariant of SARS-CoV-2 [222,223].

#### 7.3.3. mRNA Vaccines

mRNA vaccines employ a novel strategy by delivering a nucleotide sequence encoding the antigen(s) selected for their ability to elicit a protective immune response [177,224]. Although research on this technology has been going on for several years and there are potential vaccines for other infectious illnesses, the COVID-19 mRNA vaccines are the first to be approved for widespread use in public health programs [225,226]. These vaccines contain messenger RNA (mRNA) that codes for the S protein of SARS-CoV-2, the primary surface protein that binds to host cell receptors. Various approaches have been used to deliver mRNA to cells, including polymer-based nanoparticle formulation, lipid encapsulation, and incorporation of 5′-cap or 3′poly-A sequences for RNA stability and delivery. Once inside the cells, the mRNA is translated into target antigen using the cell’s machinery, which triggers an immune response [227]. Currently, four companies have produced mRNA COVID-19 vaccines, all of which are approved in at least one country.

The Comirnaty vaccine, developed by Pfizer (New York, NY, USA) and BioNTech (Mainz, Germany), has been approved in 149 countries. It comprises single-stranded, 5’ capped mRNA that encodes the SARS-CoV-2 virus’s S protein, produced through cell-free in vitro transcription using a DNA template. Clinical trials demonstrated that Comirnaty has an efficacy of 94.6% (95% CI: 89.9–97.3) against COVID-19 in adults and adolescents aged 16 and older. It also showed 100% efficacy against severe COVID-19 requiring hospitalization and COVID-19-related death. However, the emergence of new SARS-CoV-2 variants has reduced its effectiveness, prompting Pfizer and BioNTech to develop modified vaccines that include mRNA specific for the Omicron BA.1 and BA.4/BA.5 strains [228,229].

Spikevax, the mRNA COVID-19 vaccine developed by Moderna (Cambridge, MA, USA) has been approved in 88 countries. Spikevax encodes the S protein of the SARS CoV-2 virus using single-stranded, 5’ capped mRNA (known as elasomeran), which is produced via in vitro transcription on a DNA template. The mRNA is enclosed within lipid nanoparticles (LNPs), specifically SM-102, for delivery. In clinical trials, the vaccine demonstrated an efficacy of 94.1% (95% CI: 89.3–96.8) against symptomatic COVID-19 and 90.9% efficacy in patients at risk of severe COVID-19, including those with comorbidities and immunocompromised individuals [230,231].

#### 7.3.4. Viral Vector-Based Vaccines

Viral vector-based COVID-19 vaccines have garnered significant attention due to their ability to effectively deliver and express SARS-CoV-2 proteins or their epitopes as antigens [232]. These vaccines use viral vectors to carry the antigen, and they come in two varieties: non-replicating and self-replicating. After vaccination, the antigen is produced, triggering a robust humoral and cellular immune response that mimics a natural infection. Replicating vector vaccines can be administered at lower doses while still achieving a strong effect, thereby improving the safety and efficacy profiles of these vaccines [233].

The Oxford/AstraZeneca vaccine, Vaxzevria (also known as AZD1222 and ChAdOx1 nCoV-19), has been approved in 149 countries. It is made by chimpanzee adenovirus (ChAdOx1-S) that encodes the SARS-CoV-2 spike glycoprotein and is produced in genetically modified HEK 293 cells. According to studies conducted in Brazil (COV003) and the United Kingdom (COV002), the vaccine’s efficacy was approximately 60%. Another study, which included 21% of participants aged 65 and older and was carried out in the United States, Peru, and Chile, found that individuals who received two doses of the vaccine (with the second dose administered four weeks after the first) had a 74% lower incidence of symptomatic COVID-19 compared to the control group [234,235].

The Janssen (Johnson & Johnson, New Brunswick, NJ, USA) vaccine, Jcovden (also known as Ad26.COV2.S, Ad26COVS1, and JNJ-78436735), has been approved in 113 nations. It utilizes adenovirus type 26, which encodes the SARS-CoV-2 spike glycoprotein and is produced using the PER C6 TetR cell line derived from human embryonic retinal tissue. The vaccine demonstrated 66.9% efficacy after a single dose 14 days post-immunization and 66.1% efficacy 28 days post-immunization. Revised assessments indicated that the vaccine’s effectiveness against symptomatic and severe COVID-19 was 76.1% after 14 days. The booster dose administered at least two months after the initial vaccination course showed an increase in neutralizing and protein S-binding antibodies, confirming the effectiveness of booster vaccination in enhancing immunity [236,237].

The Sputnik V vaccine, also known as Gam-COVID-Vac, was developed by the Russian Gamaleya National Center of Epidemiology and Microbiology and has been approved in 75 countries. It uses a combination of two adenovirus vectors—rAD26 for the first dose and rAd5 for the second—to deliver the full-length SARS-CoV-2 S glycoprotein gene, overcoming the population’s pre-existing immunity to adenoviruses. Phase III interim findings showed that the vaccine’s efficacy was 91.6% after 21 days from the first dose and was 100% effective in preventing severe forms of COVID-19 [238,239].

With the ongoing threat of emerging variants of concern (VOCs) and variants of interest (VOIs), developing innovative vaccines that target specific alterations in viral surface proteins responsible for immune escape will be crucial for countering new variants [240]. A list of vaccines developed against SARS-CoV-2 and approved for commercialization in various countries is presented in Table 6.

## 8. Lesson(s) Learnt from These Outbreaks

RVs, such as influenza and coronaviruses, can mutate rapidly and spread globally, ultimately leading to pandemics. These pandemics are unpredictable due to antigenic drift, cross-species transmission, and the emergence of new strains and variants capable of infecting humans. Several factors contribute to the seasonality of RVs, including colder temperatures, changes in host susceptibility, and environmental conditions that favor viral spread [6]. Compared to influenza and other seasonal RVs, coronaviruses are more transmissible, causing substantial morbidity and mortality [241,242]. Furthermore, the emergence of novel variants has prolonged the duration of pandemics.

Existing antivirals, vaccines, and drugs are not always effective against all variants, and controlling viral transmission during a pandemic remains a significant challenge. To mitigate future outbreaks, new drugs with broad-spectrum antiviral activity must be developed to reduce both morbidity and mortality. Targeting highly conserved viral or host factors across all variants may inhibit viral replication and provide broad-spectrum antiviral activity against emerging viruses. Although emerging variants complicate antiviral therapy development, targeting conserved host factors during viral infection may help circumvent these challenges. However, developing therapies against host proteins requires extensive study to avoid disrupting critical physiological processes and to minimize side effects [243,244,245].

Therapeutic antibodies, such as mAbs, are effective against RV infections. However, the efficacy may be compromised by viral mutations, particularly in the receptor-binding domain, rendering some mAbs less effective against emerging variants. Broad-spectrum mAbs that target conserved viral epitopes must be developed to maintain neutralizing activity. While vaccines and antibody therapies can augment immune responses, continuous viral mutations can lead to immune evasion, reducing the effectiveness of current vaccines and therapies [246,247].

Repurposing existing drugs with proven safety profiles has emerged as a rapid strategy to address emerging RVs, as these drugs have known mechanisms of action and side-effect mitigation strategies [248]. Constant surveillance of emerging variants, antiviral efficacy, and vaccine performance is crucial to overcoming these obstacles. Given the unpredictability of future outbreaks, continuous vigilance and forecasting of pathogenic RVs are required to develop countermeasures, including broad-spectrum antivirals and vaccines [249]. Future health threats from pandemics must be addressed through risk assessments, enhanced international collaboration for epidemic and pandemic surveillance, and improved screening and diagnostics for pan-respiratory viruses.

## 9. Combating Future RV Outbreak

RVs possess a remarkable capacity to cause epidemics and pandemics due to their high mutation rates, potential for cross-species transmission, broad host range, rapid replication, contagious nature, and the lack of herd immunity in human populations. Although humans have experienced various RV pandemics and epidemics, highly effective treatment options remain limited. Understanding the biology and genomics of RVs has facilitated the development of some treatments, but these are often delayed during pandemics. Over the past two decades, influenza has caused one pandemic (swine flu, 2009), while coronaviruses have caused two epidemics (SARS, 2003; MERS, 2012) and one pandemic (COVID-19, 2019). Seasonal and climate changes continue to drive constant mutations in RVs, increasing the occurrence of cross-species infections in humans [250,251].

Recent pandemics have highlighted the immense socioeconomic burden of such outbreaks, underscoring the need for proactive antiviral development. Treatments focused on RVs that pose significant public health risks are crucial. Antiviral therapies targeting different respiratory virus genera, which vary in tissue tropism, clinical signs, and transmission profiles, are essential in preventing epidemics from escalating into pandemics. Host-directed antivirals may be particularly effective against a wide range of viruses and could prevent an epidemic from becoming a pandemic [252,253].

While annual vaccinations can control seasonal RV infections at both individual and community levels, vaccines alone cannot halt pandemics. Antigenic drift and mutations can reduce vaccine efficacy, enabling the emergence of highly virulent virus strains capable of infecting large populations. Therefore, the development of effective treatments for RVs is essential. Managing influenza and coronavirus outbreaks requires enhanced preparedness to reduce the overall burden of these infections. Key measures include prompt diagnostics, tailored treatment plans, and expanded vaccination programs targeting both coronaviruses and influenza viruses [254,255].

Proactive surveillance, combined with comprehensive mechanistic control strategies, can help manage pandemics effectively. Past outbreaks have demonstrated the need to improve techniques for characterizing antigenic properties, identifying emerging strains, and understanding the mechanisms of infection and disease pathophysiology. Developing countermeasures to protect populations vulnerable to RVs remains a critical challenge [256].

Future respiratory virus outbreaks could involve viruses that are symptomatically or antigenically similar to those of past pandemics, posing even greater threats to human populations. To effectively combat future RV pandemics and epidemics, it is essential to systematically or antigenically analyze the factors driving the outbreaks and evaluate the extent to which existing therapies can control them. Equitable vaccine distribution, public health initiatives, and the development of broad-spectrum antivirals will be crucial in continuously suppressing viral replication.

In managing pandemics, it is evident that governments and the private healthcare sector must coordinate to strengthen healthcare systems. Scientists and medical professionals worldwide must conduct comprehensive studies on pandemic pathogens using genetic monitoring and sequencing to detect and track new variants, thus developing effective antivirals, vaccines, and treatments.

International epidemiological and clinical communities must collaborate to create efficient diagnostic techniques, introduce new technologies for surveillance of emerging infections, and implement strategic public health interventions to control disease transmission. To avoid drug or vaccine shortages, proactive regulatory norms, innovative production technologies, and streamlined raw material procurement processes must be established. These initiatives, coupled with global cooperation, can mitigate the risks of future pandemics.

## Figures and Tables

**Table 1:** Comparison of influenza virus and coronavirus (SARS-CoV-2). ↓ means decreas and ↑ means increase.

Features	Influenza A Virus	SARS-CoV-2
	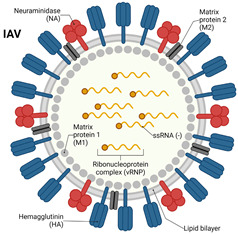	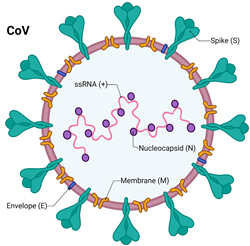
Year and pandemic name	1918 (H1N1), 1957 (H2N2), 1968 (H3N2), 2009 (H1N1), and Flu pandemics	2019 and COVID-19
Virus family	Orthomyxoviridae	Coronaviridae (genus β-CoVs)
Structure	An enveloped, negative-sense, and single-stranded RNA virus; slightly ovoid or mostly round; diameter of 80–120 nm	An enveloped, positive-sense, and single-stranded RNA virus; spherical or round in shape; diameter of 60–140 nm
Genome size	13.5 kb	29.9 kb
Mode of transmission	Droplet, aerosol, direct contact, and fecal–oral route	Droplet, aerosol, direct contact, and fecal–oral route
Replication sites	Upper respiratory tract and, in severe cases, lower respiratory tract	Starts from the upper respiratory tract, infects the lower respiratory tract, and spreads to other organs (cardiovascular, intestinal, kidney, and nervous system)
Incubation period	1–7 days	2–14 days (a maximum of 24 days)
Host receptor and entry	Terminal glycosides of sialic acid	ACE2 and TMPRSS2
Cellular tropism	Epithelial cells of Respiratory tract: Alveolar Epithelial cells and ciliated cells	Epithelial cells of the respiratory tract: alveolar epithelial cells, ciliated cells, basal cells of the olfactory epithelium, intestinal epithelial cells, renal parenchymal cells, and endothelial cells
Viral protein binding to host receptor	HA	Spike (S) protein
Replication	Nuclear	Cytoplasm
Symptom	Fever, dry cough, sore throat, fatigue, and nasal congestion	High fever, dry cough, fatigue, ARDS, and anosmia
Extrapulmonary complications	In rare cases, myocarditis and encephalitis	In most cases, anosmia, thrombosis, stroke, encephalitis, and diarrhea
Target for neutralizing antibodies	HA and NA	RBD of the spike protein
Hematological parameters	Lymphopenia and CRP ↑	Type I interferon ↓, neutrophil counts ↑, and significant lymphopenia
Variants of concern (VOCs)	1957 H2N2, 1968 H3N2, and 2009 H1N1	Alpha, Beta, Gamma, Delta, and Omicron
Mortality rate	0.05–0.1% (seasonal influenza)	~1–3.4% (higher in early wave)
Vaccine availability	Annual seasonal vaccines (inactivated, live)	Multiple vaccines (mRNA, vector-based, inactivated)
Mutations/variants	Antigenic shift and drift	Frequent mutations with variants of concern (e.g., Delta and Omicron)
Treatment options	Antivirals (e.g., oseltamivir and zanamivir)	Antivirals (e.g., remdesivir, molnupiravir, and Paxlovid), mAbs
Complications	Pneumonia and secondary bacterial infections	Pneumonia, acute respiratory distress syndrome (ARDS), and multi-organ damage
References	[26,27]	[28,29]

**Table 4:** Clinical trials on treatments for RV infections.

Drug	Virus Target	Clinical Trial Identifier	Phase	Classification	Function
**Camostat mesylate**	Influenza virus A/B/SARS-CoV-2	NCT04470544	II	TMPRSS2 serine protease inhibitor	Blocking the virus activating host cell protease TMPRSS2
**Baloxavir marboxil**	Influenza virus A/B	NCT03684044	III	Polymerase acidic (PA) endonuclease inhibitor	Inhibits viral replication
**Pimodivir**	Influenza virus A/B	NCT02262715 NCT02342249	I II	PB2 inhibitor	Inhibits viral replication
**Enisamium iodide**	Influenza virus A/B	NCT04682444 NCT04682873	II/III III	RNA polymerase inhibitor	Inhibits viral replication
**DAS181**	Influenza virus A/B	NCT01173224 NCT01651494 NCT00527865 NCT01037205	I	Entry inhibitor	Removes sialic acid from epithelial cells, preventing viral entry
**Remdesivir**	SARS-CoV-2	NCT04345419 NCT04678739	II/III	RdRp inhibitor	Inhibit viral RNA replication by binding with viral RNA
**Favipiravir**	SARS-CoV-2	NCT04303299 NCT04351295 NCT04600999 NCT04346628 NCT04387760 NCT04542694 NCT03394209	III II/III III II II III II	RdRp inhibitor	Inhibits viral replication and genetic transversion
**Ribavirin**	SARS-CoV-2	NCT01097395 NCT01497366 NCT04276688 NCT04563208	IV III II II	RdRp inhibitor	Inhibits viral RNA synthesis and immunomodulation
**Ivermectin**	SARS-CoV-2	NCT04403555 NCT04381884 NCT04646109 NCT04591600	II/III II III I/II	Viral protease inhibitor	Inhibits viral protein transport to nucleus
**Ritonavir**	SARS-CoV-2	NCT04303299	III	Viral protease inhibitor	Inhibits viral Plpro protease activity
**Lopinavir**	SARS-CoV-2	NCT04276688 NCT04252885	II IV	Viral protease inhibitor	Inhibits viral 3CLpro activity
**Eculizumab**	SARS-CoV-2	NCT04346797	II	Monoclonal antibody	Prevents the activation of inflammation by inhibiting the C5 complement protein
**Bevacizumab**	SARS-CoV-2	NCT04275414	II	Monoclonal antibody	Preventing acute lung injury in ARDS and suppression of pulmonary edema
**Meplazumab**	SARS-CoV-2	NCT04275245	II/III	Monoclonal antibody	Prevents viral entry and inflammation
**Ramipril**	SARS-CoV-2	NCT04366050	II	ACE inhibitor	Ras inhibitor to stop heart failure
**Azithromycin**	SARS-CoV-2	NCT04332107 NCT04381962	III III	RNA inhibitor	Regulates cytokine storm
**Chloroquine**	SARS-CoV-2	NCT04420247 NCT04353336	III II/III	Derivatives of quinine compounds	Inhibit virion formation and MAPK activation
**Colchicine**	SARS-CoV-2	NCT04322682 NCT04472611	III III	Anti-inflammatory drug	Inactivates pro-inflammatory cytokines and migration of leukocytes
**Baricitinib**	SARS-CoV-2	NCT04421027 NCT04373044	II/III II	JAK1/AAK1 inhibitor	Suppresses inflammatory factors (IL-6 and IL7)
**Methylprednisolone**	SARS-CoV-2	NCT04244591 NCT04263402 NCT04273321	II/III/IV	Corticosteroid	Lowers the viral lung damage
**APN01-COVID-19**	SARS-CoV-2	NCT04335136	II	Recombinant human angiotensin-converting enzyme 2 (rhACE2)	Prevents viral entry and viral replication

**Table 5:** Summary of approved vaccines for the treatment of infections caused by influenza viruses.

Vaccine Name	Manufacturer	Vaccine Type	Approved Country
**Influgen**	Lupin Laboratories Ltd., Mumbai, India	Inactivated influenza vaccine	India
**Fluzone Quadrivalent**	Sanofi Pasteur, Inc., Swiftwater, PA, USA	Inactivated influenza vaccine	USA
**FluQuadri**	Sanofi-Aventis, Macquarie Park, Australia	Inactivated influenza vaccine	Australia
**Vaxigrip Tetra**	Sanofi-Aventis, Macquarie Park, Australia	Inactivated influenza vaccine	Australia
**Fluarix Tetra**	Glaxo-SmithKline Biologicals, Rixensart, Belgium	Inactivated influenza vaccine	Australia
**Afluria Quadrivalent**	Seqirus-Pty. Ltd., Parkville, Australia	Inactivated influenza vaccine	USA
**Fluarix Quadrivalent**	Glaxo-SmithKline Biologicals	Inactivated influenza vaccine	USA
**Fluad Quadrivalent**	Seqirus, Inc., Holly Springs, NC, USA	Inactivated influenza vaccine	USA
**Agripal**	Chiron Panacea Vaccines Pvt. Ltd., New Delhi, India	Inactivated influenza vaccine	India
**Influvac Tetra**	Mylan Health, Canonsburg, PA, USA	Inactivated influenza vaccine	Australia
**FluLaval-Quadrivalent**	ID-Biomedical, Laval, QC, Canada	Inactivated influenza vaccine	USA
**FlublokQuadrivalent**	Sanofi Pasteur, Inc.	Recombinant influenza vaccine	USA
**Cadiflu-S Vaccine**	CPL Biologicals Pvt Ltd., Ahmedabad, India	Inactivated influenza vaccine	India
**FluMist-Quadrivalent**	Med-Immune, LLC., Gaithersburg, MD, USA	Live attenuated influenza vaccine	USA
**Flucelvax-Quadrivalent**	Seqirus, Inc.	Inactivated influenza vaccine	USA
**Nasovac S Vaccine**	Serum Institute of India Ltd., Pune, India	Inactivated influenza vaccine	India

**Table 6:** Summary of approved vaccines for the treatment of COVID-19.

Types of Vaccine	Vaccine Name	Produced by	Approval Status	Trials	No. of Countries Allowed Trials	Approved Countries
Inactivated vaccine	Covaxin	Bharat Biotech, Hyderabad, India	Approved	16	2	14
Physically or chemically inactivated viral vaccines	KoviVac	Chumakov Center, Moscow, Russia	Approved	5	1	3
	Turkovac	Health Institutes of Turkey, Ankara, Turkey	Approved	8	1	1
	FAKHRAVAC (MIVAC)	Organization of Defensive Innovation and Research, Tehran, Iran	Approved	3	1	1
	QazVac	Research Institute for Biological Safety Problems (RIBSP), Gvardeyskiy, Kazakhstan	Approved	3	1	2
	KCONVAC	Shenzhen Kangtai Biological Products Co., Shenzhen, China	Approved	7	1	2
	COVIran Barekat	Shifa Pharmed Industrial Co, Tehran, Iran	Approved	6	1	1
	Covilo	Sinopharm, Beijing, China	Approved	39	18	93
	Inactivated (Vero Cells)	Sinopharm, Wuhan, China	Approved	9	7	2
	CoronaVac	Sinovac, Beijing, China	Approved	42	10	56
	SKYCovione	SK Bioscience Co. Ltd., Seongnam, South Korea	Approved	7	6	1
	VLA2001	Valneva, Saint-Herblain, France	Approved	9	4	33
Protein subunit vaccines	Zifivax	Anhui Zhifei Longcom, Hefei, China	Approved	21	5	4
Use a protein fragment or viral spike proteins as the antigen to trigger an immune response	Noora vaccine	Bagheiat-allah University of Medical Sciences, Tehran, Iran	Approved	3	1	1
	Corbevax	Biological E Limited, Hyderabad, India	Approved	7	1	2
	Soberana 02	Instituto Finlay de Vacunas, Havana, Cuba	Approved	7	2	4
	Soberana Plus	Instituto Finlay de Vacunas	Approved	5	1	2
	V-01	Livzon Mabpharm Inc., Zhuhai, China	Approved	7	3	1
	MVC-COV1901	Medigen, Taipei, Taiwan	Approved	15	4	4
	Recombinant SARS-CoV-2 Vaccine (CHO Cell)	National Vaccine and Serum Institute, Beijing, China	Approved	3	2	1
	Nuvaxovid	Novavax, Gaithersburg, MD	Approved	22	14	40
	IndoVac	PT Bio Farma, Bandung, Indonesia	Approved	4	1	1
	Razi Cov Pars	Razi Vaccine and Serum Research Institute, Karaj, Iran	Approved	5	1	1
	VidPrevtyn Beta	Sanofi/GSK, Lyon, France/Brentford, UK	Approved	3	2	30
	COVOVAX	Serum Institute of India, Pune, India	Approved	7	3	6
	TAK-019 (Novavax formulation)	Takeda, Tokyo, Japan	Approved	3	1	1
	SpikoGen	Vaxine/CinnaGen Co., Adelaide, Australia	Approved	8	2	1
	Aurora-CoV	Vector State Research Center of Virology and Biotechnology, Koltsovo, Russia	Approved	2	1	1
	EpiVacCorona	Vector State Research Center of Virology and Biotechnology	Approved	4	1	4
Non-replicating viral vector vaccines	iNCOVACC	Bharat Biotech	Approved	4	1	1
Bioengineered viral vectors that are unable to express and clone antigens originating from the targeted virus	Convidecia	CanSino, Tianjin, China	Approved	14	6	10
	Convidecia Air	CanSino	Approved	5	4	2
	Abdala	Center for Genetic Engineering and Biotechnology (CIGB), Havana, Cuba	Approved	5	1	6
	Gam-COVID-Vac	Gamaleya, Moscow, Russia	Approved	2	-	1
	Sputnik Light	Gamaleya	Approved	7	3	26
	Sputnik V	Gamaleya	Approved	25	8	74
	Jcovden	Janssen (Johnson & Johnson), New Brunswick, NJ	Approved	26	25	113
	Vaxzevria	Oxford/AstraZeneca, Oxford, UK	Approved	73	34	149
	Covishield	Serum Institute of India	Approved	6	1	49
RNA vaccines	GEMCOVAC-19	Gennova Biopharmaceuticals Limited, Pune, India	Approved	2	1	1
Vaccines based on mRNA that are combined with injectable nanoparticles to efficiently transfer the mRNA into target cells and induce adaptive immunity	Spikevax	Moderna, Cambridge, MA, USA	Approved	70	24	88
	Spikevax Bivalent Original/Omicron BA.1	Moderna	Approved	5	4	38
	Spikevax Bivalent Original/Omicron BA.4/BA.5	Moderna	Approved	2	1	33
	Comirnaty	Pfizer/BioNTech, New York, NY, USA/Mainz, Germany	Approved	100	31	149
	Comirnaty Bivalent Original/Omicron BA.1	Pfizer/BioNTech	Approved	3	5	35
	Comirnaty Bivalent Original/Omicron BA.4/BA.5	Pfizer/BioNTech	Approved	4	1	33
	TAK-919 (Moderna formulation)	Takeda	Approved	2	1	1
	AWcorna	Walvax, Kunming, China	Approved	4	3	1
DNA vaccines	ZyCoV-D	Zydus Cadila, Ahmedabad, India	Approved	6	1	1
DNA vaccines comprise of a plasmid that has been genetically modified to encode the antigen unique to the disease and a vector that transports the plasmid into the host cell.						
VLP vaccines	Covifenz	Medicago, Quebec City, QC, Canada	Approved	6	6	1
Spontaneously assembled from several structural proteins of the virus to stimulate the immune system						

## Data Availability

The data are available from the corresponding author upon reasonable request.
